# Constraints on Exchange Edits During Noisy‐Channel Inference

**DOI:** 10.1111/cogs.70143

**Published:** 2025-11-25

**Authors:** Markus Bader, Michael Meng

**Affiliations:** ^1^ Institute of Linguistics Goethe University Frankfurt; ^2^ Department of Business Administration and Information Sciences University of Applied Sciences Merseburg

**Keywords:** Sentence processing, Noisy channel, Rational inference, Psycholinguistics, German

## Abstract

According to the noisy channel framework of sentence processing, communication can succeed even when the input is corrupted because comprehenders rationally infer the speaker's intended meaning based on the prior probability of the literal interpretation and the probability that the input has been corrupted by noise. To test whether and under what conditions comprehenders consider word exchanges as a possible source of corruption, we ran five experiments on processing three types of simple German sentences: subject‐before‐object sentences (SO), object‐before‐subject sentences (OS), and passive sentences. Critical sentences had implausible meanings, but could be “repaired” by exchanging function words or by exchanging nouns. Experiments 1 through 4 presented sentences along with yes‐no questions to probe interpretation. Implausible SO and passive sentences consistently elicited few nonliteral interpretations, whereas many nonliteral interpretations were given to implausible OS sentences. This was true regardless of whether word exchanges had to cross a main verb or an auxiliary, and it was more pronounced if the overall proportion of implausible sentences was low. We conclude that when answering yes‐no questions, word exchanges are considered with function words of the same syntactic category, but not with nouns, and only when they result in a more likely syntactic structure. Experiment 5 showed that when explicitly asked to correct implausible sentences, comprehenders use noun exchanges frequently. We propose that the results for both yes‐no questions and explicit corrections follow if the prior probability assigned to implausible sentences differs between tasks.

## 1 Introduction

Language comprehension is a seemingly effortless everyday activity. Nevertheless, systematic error patterns have been observed in experiments that tested language comprehension in different languages and constructions, using different tasks to assess final sentence interpretation. For example, participants often make errors when asked to identify the agent or patient of implausible passive sentences such as *The dog was bitten by the man* (Christianson et al., 2010; Ferreira, 2003). Likewise, several studies demonstrated that participants often interpret sentences like *The mother gave the candle the daughter* as meaning that the daughter received the candle (Buxó‐Lugo & Slevc, 2023; Cai et al., 2022; Gibson et al., 2013; Poppels & Levy, 2016).

While the existence of nonliteral interpretations has been firmly established, their source remains a controversial issue. One dimension along which accounts differ is whether nonliteral interpretations are assumed to reflect errors in the mapping of form to meaning (Dempsey et al., 2023; Karimi & Ferreira, 2016), or whether errors are assumed to be post‐interpretive and induced by task‐specific processes that operate on sentence representations encoding the correct interpretation (Bader and Meng, 2018; Meng & Bader, 2025). A second dimension differentiating current accounts concerns the processing mechanisms giving rise to nonliteral interpretations. For example, the good enough framework of sentence comprehension assumes processing mechanisms that are designed to provide a fast route to interpretation. This avoids complex computations, but can lead to errors when more complex computations are required to arrive at the correct interpretation (Ferreira & Patson, 2007; Karimi & Ferreira, 2016). The current paper focuses on an alternative proposal, the Noisy Channel Model (Gibson et al., 2013; Levy, 2008), which views nonliteral interpretations not as errors but as the result of rational inferences which take into account that the input to the language comprehension mechanisms may be distorted for a variety of reasons, including speech errors, background noise, and slurred speech.

The question of how communication can succeed despite imperfections in the input has become a major topic of research, inspired by the work of Levy, Gibson, and colleagues on rational language comprehension (Chen et al., 2023; Gibson et al., 2013; Levy, 2008; Poliak et al., 2024; Poppels & Levy, 2016; Ryskin et al., 2025). According to the Noisy Channel Model of Gibson et al. (2013), comprehenders entertain alternative hypotheses about what the speaker intended. Using probabilistic inferences, interpreters estimate how likely possible intended sentences $s_{i}$ are for a perceived sentence $s_{p}$, as expressed by the conditional probability $P(s_{i}|s_{p})$ on the left side of the formula below:


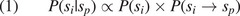


Following Bayes' rule, the language comprehender is hypothesized to estimate $P(s_{i}|s_{p})$ from the two probabilities given on the right‐hand side of the formula: the prior probability $P(s_{i})$ of producing the intended sentence $s_{i}$ and the conditional probability $P(s_{i}\to s_{p})$ that $s_{i}$ is perceived by the hearer/reader as $s_{p}$.^1^

The prior probability $P(s_{i})$ reflects, among other things, the plausibility of the meaning associated with $s_{i}$ and the frequency of the grammatical structure of $s_{i}$. The second term $P(s_{i}\to s_{p})$ represents the comprehender's noise model, which captures the probability that the input has been corrupted by noise. It is estimated by taking into account how extensive the possible corruption is and to which extent such a corruption can be expected in the given communicative environment. The final interpretation adopted by the language comprehender corresponds to the intended sentence $s_{i}$ that maximizes $P(s_{i}|s_{p})$.

The evaluation process assumed by the Noisy Channel Model biases comprehenders toward plausible sentences with frequent syntactic structures. In most cases, $s_{i}$ is identical to $s_{p}$, in which case the comprehender adopts the meaning corresponding to the input sentence $s_{p}$. Sometimes, however, a meaning will be adopted that does not correspond to $s_{p}$. When the prior probability of $s_{p}$ is low because its meaning is implausible and/or its syntactic structure is of low frequency, and there is an alternative sentence $s_{i}$ that is more plausible and/or of higher structural frequency, the comprehender may conclude that the intended sentence $s_{i}$ was corrupted and that the producer in fact intended $s_{i}$ instead of $s_{p}$.

Importantly, the availability of an alternative $s_{i}$ with higher prior probability than the perceived sentence $s_{p}$ is not sufficient to discard $s_{p}$ in favor of $s_{i}$. For this to happen, the comprehender must consider it likely that the hypothesized intended sentence $s_{i}$ got corrupted to the perceived sentence $s_{p}$, as captured by the term $P(s_{i}\to s_{p})$. Whether an $s_{i}$ different from $s_{p}$ is indeed adopted depends on the gain in prior likelihood compared to the likelihood of the “edit” operations necessary to convert $s_{i}$ to $s_{p}$. The higher the gain in prior probability due to a repair, the more extensive the hypothesized edits can be.

The predictions of the Noisy Channel Model have been tested on a range of different syntactic structures. Most studies used a paradigm that required participants to answer a yes‐no comprehension question for each sentence they read (e.g., Chen et al., 2023; Gibson et al., 2013, 2017; Poppels & Levy, 2016; Poliak et al., 2024; Zhan et al., 2023; Ryskin et al., 2021, 2025). Of particular importance for the development of the Noisy Channel Model of Gibson et al. (2013) were highly implausible double object (DO) and prepositional object (PO) sentences as in (2‐a) and (2‐b).


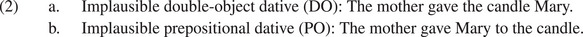


When asked questions like “Did the candle receive something/someone?” or “Did Mary receive something/someone?”, participants in Experiment 1 of Gibson et al. (2013) gave the correct answer in about 60% of all cases for implausible PO sentences and in about 50% for implausible DO sentences. These rather low accuracy values contrast sharply with the accuracy values of nearly 100% for corresponding plausible sentences (*The mother gave Mary the candle./The mother gave the candle to Mary*.). The Noisy Channel Model accounts for this effect because the implausible sentences compete with close variants that have a plausible meaning and can be derived from the actual sentences by assuming that the preposition *to* got deleted (cf. (2‐a)) or inserted (cf. (2‐b)).

### 1.1 Constraints on word exchanges

The focus of the current paper is the noise model $P(s_{i}\to s_{p})$, which estimates what edit operations comprehenders consider as likely. Possible edit operations are deletions and insertions of one or more words, as well as positional exchanges of words. Because high rates of nonliteral interpretations were found for implausible DO and PO sentences as in (2), Gibson et al. (2013) concluded that comprehenders take single deletions and insertions into account when forming the set of potentially intended sentences $s_{i}$. In contrast to implausible DO and PO sentences, participants in Gibson et al.'s experiments interpreted simple active and passive sentences rarely, if at all, in a nonliteral way even when they were highly implausible, as the examples in (3).


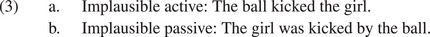


Implausible active and passive sentences as in (3) can be derived from plausible counterparts in several ways. For example, the speaker may have intended the sentence *The ball was kicked by the girl*, but due to noise the two words *was* and *by* were deleted and the hearer got as input the implausible active sentence in (3‐a). Alternatively, the speaker may have intended the sentence *The girl kicked the ball*., but due to noise (e.g., a slip of the tongue), the two nouns got exchanged, again resulting in the implausible active sentence (3‐a). In a similar way, the implausible passive sentence (3‐b) can result if the speaker intended the sentence *The girl kicked the ball*, but due to noise the two words *was* and *by* were inserted, or the speaker intended *The ball was kicked by the girl* but the nouns got exchanged. Since implausible active and passive sentences elicited few nonliteral interpretations if at all, Gibson et al. (2013) concluded that comprehenders consider all edit operations that go beyond single insertions or deletions as so unlikely that the gain in plausibility is not large enough to outrank the literal interpretation. Poppels & Levy (2016) took issue with this conclusion by noting that the finding of few nonliteral interpretations for implausible active and passive sentences as in (3) does not necessarily mean that comprehenders consider all kind of word exchanges as highly unlikely. Instead, Poppels & Levy raise the possibility that only certain types of exchanges are banned from consideration. They discuss two possible constraints on exchange edits: that word exchanges are restricted to function words (henceforth referred to as Function Word Constraint, see (4)) and that word exchanges affect words in arguments, but not in adjuncts (henceforth referred to as Adjunct Constraint, see (5)).


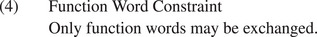



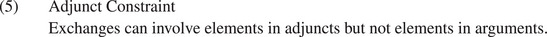


Both constraints exclude noun exchanges in simple implausible active and passive sentences. The Function Word Constraint excludes them because nouns are not function words. The Adjunct Constraint excludes them because the exchanges affect arguments and not adjuncts.

In order to distinguish between a general constraint prohibiting all kinds of word exchanges and more specific constraints as in (4) and (5), Poppels & Levy (2016) investigated the comprehension of double PP sentences as in (6).


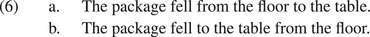


An implausible input sentence like (6‐a) results from an intended plausible sentence like *The package fell to the floor from the table* by exchanging prepositions, which is allowed by both the Function Word Constraint and the Adjunct Constraint but not by a general constraint banning word exchanges. An implausible input sentence like (6‐a) also results if nouns are exchanged in a plausible sentence like *The package fell from the table to the floor*, which is allowed under the Adjunct Constraint but not under the Function Word Constraint. Analogous considerations apply to sentence (6‐b). Poppels & Levy (2016) found that the frequency of nonliteral interpretations was substantially higher for implausible double PP sentences than for implausible active or passive sentences, although not as high as for implausible DO and PO sentences. From these findings, Poppels & Levy (2016) conclude that comprehenders take word exchanges into account when drawing noisy‐channel inferences, subject to constraints like the Function Word Constraint or the Adjunct Constraint. Differentiating between these two constraints was not possible on the basis of the data obtained by Poppels & Levy (2016). Zhan et al. (2023) proposed another potential restriction on word exchanges, which will be referred to as the *Intervening Main Verb Constraint*, paraphrased in (7).


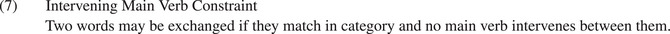


The Intervening Main Verb Constraint blocks exchanges in implausible active and passive sentences but allows them in double PP sentences (see also Chen et al., 2023). Evidence for the Intervening Main Verb Constraint derives from experimental work on Chinese active and passive sentences reported in Zhan et al. (2023). An example is provided below (example (5) of Zhan et al., 2023; for reason of space, only English glosses and translations are given).


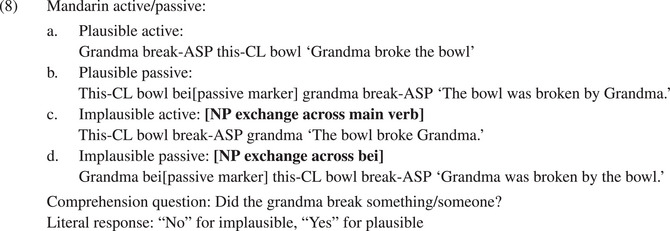


As in English, implausible active and passive sentences can be turned into plausible sentences by exchanging the nouns of subject NP and nonsubject NP. In English, this exchange crosses a main verb in both active and passive sentences and is, therefore, regarded unlikely according to the Intervening Main Verb Constraint. In Mandarin Chinese, an asymmetry arises: restoring an implausible active sentence requires an NP exchange across an intervening main verb, whereas an implausible passive sentence can be restored by an NP exchange across the passive marker “bei.” This operation reverses the thematic roles of the NPs, resulting in a plausible sentence. Using the same yes‐no question procedure as Gibson et al. (2013), Zhan et al. (2023) found a higher rate of nonliteral interpretations for implausible passive compared to implausible active sentences.

### 1.2 The present study

In order to decide between the competing constraints on exchange edits, we ran a series of experiments that investigated the conditions under which nonliteral interpretations are observed in simple German main clauses. As in English, a canonical main clause in German realizes the agent argument as subject, the patient argument as object, and the subject/agent precedes the object/patient. This is illustrated in (9).


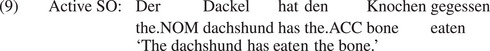


As can be seen in (9), whereas English has only a single determiner *the* for subjects and objects, German has distinct determiners, *der* for subjects and *den* for objects. Thus, both determiner exchanges and noun exchanges have visible effects on German main clauses. In contrast to English, possible differences between function words and content words with regard to word exchanges can be revealed by investigating simple main clauses.

Noncanonical sentences in German can deviate from canonical sentences in two ways. First, the object can be placed in front of the subject, resulting in an object‐before‐subject (OS) sentence as in (10). In OS sentences, both the order of syntactic functions (obj < subj) and the order of thematic roles (patient < agent) is noncanonical. Note that OS sentences are grammatical, but marked in the sense that they require certain discourse conditions to be fully acceptable (see Bader & Meng, 2023; Weskott et al., 2011 for discussion and further references).


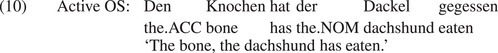


Noncanonical sentences can also be created by passivization, which promotes the patient argument to the subject function and demotes the agent argument to a *by*‐phrase. As illustrated in (11), passive sentences vary with regard to word order in a similar way as active sentences. A passive sentence can start with the subject, in which case the order of agent and patient is noncanonical, as in (11‐a). Alternatively, a passive sentence can start with the *by*‐phrase, as in (11‐b), resulting in a noncanonical order of syntactic functions combined with a canonical order of thematic roles.


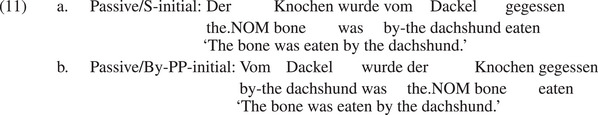


As discussed above, the prior probability $P(s_{i})$ of producing an intended sentence $s_{i}$ depends both on the meaning and the syntactic structure of the sentence. To estimate the contribution of the structure of $s_{i}$ to $P(s_{i})$, Table 1 shows how often the four syntactic structures introduced above occur in the Tiger 2.1 treebank (https://www.ims.uni‐stuttgart.de/forschung/ressourcen/korpora/tiger/). In accordance with their status as canonical sentences, active SO sentences are by far the most common. Of the four alternative realizations of a sentence with an agent and a patient, over 80% are active SO sentences. Active OS sentences are much less frequent, but they still occur with some regularity. Passive sentences are, in general, less frequent than active sentences, but similar to active sentences, subject‐initial passive sentences are much more frequent than nonsubject initial passive sentences.

**Table 1:** Frequency counts of the four syntactic structures investigated in this study.

	Active SO	Active OS	Passive/Subj‐first	Passive/PP‐first
Frequencies	4462	652	223	19
Percentages	83.31%	12.17%	4.16%	0.35%

*Note*. Data are taken from the Tiger 2.1 treebank. Active sentences include all main clauses containing at least a subject and an accusative object. Passive sentences include all main clauses containing at least a subject and a by‐PP.

Next to structural frequency, plausibility is the second major determinant of prior probability. Following much research on noisy‐channel inference (e.g., Gibson et al., 2013; Poppels & Levy, 2016), our experiments manipulate plausibility in a coarse‐grained manner by contrasting sentences that describe events that are possible according to general world‐knowledge (e.g., a dog eating a bone) with sentences that are impossible (e.g., a bone eating a dog). Also, following research on noisy‐channel inference, we will call sentences of the former type “plausible” and sentences of the latter type “implausible” (in other areas of research, such sentences are called “anomalous”; e.g., Rayner et al., 2004).

The prior probability is highest for sentences that are plausible and have a frequent syntactic structure, that is, plausible SO sentences in the current context. All other sentence types—implausible SO sentences as well as plausible and implausible noncanonical sentences—have a reduced prior probability and, therefore, compete with sentences of higher prior probability. For these sentences, comprehenders may adopt a nonliteral interpretation depending on the gain in prior probability and the particular noise model.

Regarding noisy channel inferences, we first consider implausible SO sentences as well as plausible and implausible OS sentences. Passive sentences will be considered in more detail in the introduction to Experiment 2. Implausible OS sentences have the lowest prior probability because they are both semantically and syntactically improbable. An example is provided in (12).


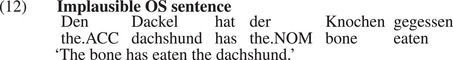


Suppose that someone is reading sentence (12). On recognizing that this is a highly implausible and thus highly unlikely sentence, the reader may reason that the writer did not intend this sentence but a plausible sentence instead in which the dachshund is the subject and the bone is the object. Depending on the syntactic structure of the intended plausible sentence, either a determiner or a noun exchange will result in the implausible sentence (12). One possibility is that the writer intended the plausible SO sentence *Der Dackel hat den Knochen gegessen* (“The dachshund has eaten the bone”), but exchanged the determiners of subject and object, as illustrated in (13).


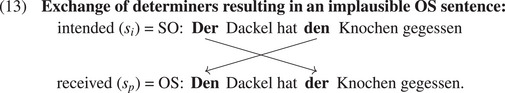


Alternatively, the writer may have intended the plausible OS sentence *Den Knochen hat der Dackel gegessen* (“The bone, the dachshund has eaten”) but exchanged nouns, as illustrated in (14).


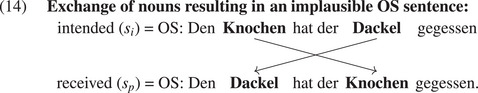


A determiner exchange changes the meaning and the syntactic structure of a sentence. The intended sentence in the case of a determiner exchange is a plausible SO sentence, which is more likely than the perceived implausible OS sentence both semantically and syntactically. A noun exchange, in contrast, does not alter the syntactic structure of the sentence. The intended plausible OS sentence is thus only semantically more likely than the perceived implausible OS sentence.

Like implausible OS sentences, implausible SO sentences may cause the reader to reason that the writer intended a plausible sentence instead. Consider the example in (15), which is the SO counterpart to sentence (12).


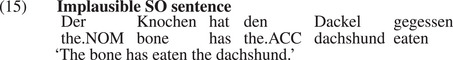


Similar to implausible OS sentences, implausible SO sentences can be the result of either a determiner or a noun exchange. If the writer intended a plausible OS sentence but mistakenly mixed up the two determiners, an implausible SO sentence results, as shown in (16).


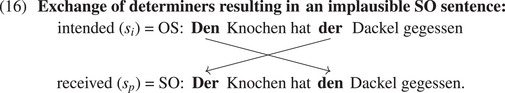


Alternatively, the writer may have intended a plausible SO sentence but then exchanged the two nouns, resulting in an implausible SO sentence, as shown in (17).


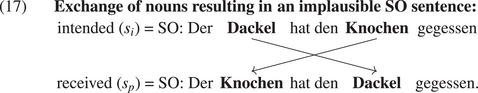


To sum up the discussion so far, the plausibility of both implausible SO and OS sentences can be restored equally in two ways: by assuming noun exchanges or by assuming function word (i.e., determiner) exchanges. With regard to the gain in prior probability, however, there is a crucial difference between implausible SO and implausible OS sentences depending on whether noun exchanges or function word exchanges are assumed. When implausible sentences are restored to plausibility by means of a noun exchange, the intended and the perceived sentence share the same syntactic structure—an intended SO sentence is perceived as SO sentence and an intended OS sentence is perceived as OS sentence. Thus, under the assumption that the noise model favors noun exchanges, the rate of nonliteral interpretation should be the same for implausible SO and OS sentences because the gain in prior probability for the intended plausible sentence is identical for both, and without a change of syntactic structure, prior probability is not affected by structural frequency.

In the case of a determiner exchange, in contrast, the likelihood of interpreting implausible SO and OS sentences in a nonliteral way is predicted to differ because a determiner exchange reverses the order of subject and object and SO and OS sentences have different prior probabilities. As shown in Table 1, SO sentences are about 6.8 times more frequent than OS sentences. Thus, when considering a plausible SO sentence as the underlying source of an implausible OS sentence, as in (16), the plausible SO variant is more likely in terms of meaning and structure. For a plausible OS sentence as an alternative to an implausible SO sentence, as in (13), in contrast, the OS sentence is more likely with respect to meaning but less likely with regard to structure. We have no corpus data on the frequency of highly implausible sentences like *The bone ate the dachshund*, but it seems safe to assume that such sentences are produced with the implausible meaning being the intended meaning with a frequency of less than 1%. Thus, the prior probability of a plausible OS sentence will still be higher than the prior probability of an implausible SO sentence, although the gain in probability will not be as high as in the reverse case (plausible SO compared to implausible OS).

In sum, the predicted rates of nonliteral interpretations for implausible SO and implausible OS sentences differ depending on whether noun or determiner exchanges are ranked higher in the noise model of the reader. When an implausible sentence is edited by a noun exchange, the syntactic structure does not change. Since the gain in plausibility is the same for SO and OS sentences, this implies that the rate of nonliteral interpretations should be the same for SO and OS sentences. On the other hand, an exchange of determiners causes a switch of the syntactic structure. Since a switch from SO to OS results in a less probable structure, whereas a switch from OS to SO results in a more probable structure, more nonliteral interpretations should be observed for implausible OS than for implausible SO sentences. We thus get the following predictions.


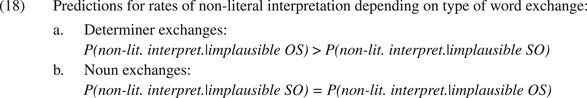


The final sentence type to consider is represented by plausible OS sentences as in (10), repeated below for convenience.


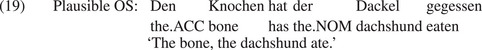


Because plausible OS sentences have a lower prior probability than plausible SO sentences, a reader perceiving a plausible OS sentence may reason that the writer intended a plausible SO sentence. In this case, the writer must have exchanged both determiner and noun, because exchanging only one would result in an implausible sentence, which has a much lower prior probability. Because the gain in prior probability is only mild in this case and the probability of two exchange errors occurring simultaneously is low, we consider it as unlikely that readers will adopt nonliteral interpretations in this case. Note furthermore that, due to a lack of semantic difference between intended and perceived sentence, such nonliteral interpretations cannot be detected with yes‐no comprehension questions, which we will use in our experiments following a widespread practice in research on the Noisy Channel Model (e.g., Gibson et al., 2013; Poppels & Levy, 2016).

Because German is a verb‐second language, it allows a direct test of the Intervening Main Verb Constraint. As a verb‐second language, German places the finite verb in second position in main clauses. With a composite tense form as in (12) and (15), the finite verb is an auxiliary, which should not block word exchanges according to the Intervening Main Verb Constraint. With a noncomposite tense, in contrast, the main verb itself is the finite verb, which thus intervenes between subject and object, as shown in (20) for an implausible SO sentence and in (21) for an implausible OS sentence.


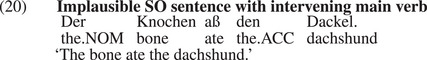



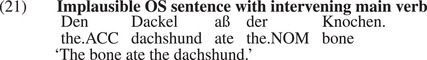


According to the Intervening Main Verb Constraint, comprehenders should not consider the possibility that (20) and (21) resulted from word exchanges applied to plausible sentences. Thus, this hypothesis predicts a fundamental difference between sentences with composite and sentences with non‐composite tense form. Finally, the Adjunct Constraint predicts that comprehenders should not consider the possibility of word exchanges for restoring the plausibility of implausible SO and OS sentences at all, since subject and object are not adjuncts and should, therefore, not be affected by word exchanges. Table 2 summarizes the predictions that follow from the three constraints under discussion.

**Table 2:** Summary of the predictions for implausible German SO and OS main clauses with main verb (MV) or auxiliary verb (Aux) intervening between subject and object.

Constraint	Edit operation(s)	S‐Aux‐O	S‐MV‐O	O‐Aux‐S	O‐MV‐S
No constraint	Noun or Det Exchanges	√	√	√	√
Function Word Constraint	Det Exchanges	−	−	√	√
Adjunct Constraint	Noun or Det Exchanges	−	−	−	−
Intervening Main Verb Constraint	Noun or Det Exchanges	√	−	√	−

*Note*. Conditions for which an increased rate of nonliteral interpretations is expected are marked by “√.”

The remainder of the paper is organized as follows. The predictions that the three constraints discussed above make for exchange edits in implausible German SO and OS sentences are tested in Experiment 1. The conclusions reached from Experiment 1 are corroborated and extended in Experiment 2 and Experiment 3. Experiment 2 compares plausible and implausible SO and OS sentences to plausible and implausible passive sentences. This provides an additional test case to answer the question whether restoring the plausibility of implausible sentences involves exchanges of function words or exchanges of nouns. Experiment 3 tests whether the likelihood of nonliteral interpretations of OS sentences is affected by the prior probability $P(s_{i})$, as predicted by the Noisy Channel Model. Following Gibson et al. (2013), the prior probability $P(s_{i})$ is varied by varying the total rate of implausible sentences that participants encounter in an experimental session. Experiment 4 tests an alternative account of the results of Experiments 1–3 in terms of edit operations at the letter level. Finally, Experiment 5 complements the yes‐no comprehension task used in the first four experiments with an explicit correction task. Following up on Ryskin et al. (2018), Experiment 5 addresses whether the constraints on word exchanges established in Experiments 1–4 are dependent on the task used to assess comprehension.

## 2 Experiment 1

Experiment 1 examined the interpretation of simple German main clauses consisting of a subject, one or two verbs, and an object. As illustrated by the sentences in (12), (15), (20), and (21) above (see also Table 3 for a complete stimulus item), sentences vary according to three factors: the factor *Plausibility* varies whether sentences are plausible or implausible; the factor *Order* varies whether sentences occur with SO or OS order; the factor *Verb* varies whether an auxiliary or a main verb occurs between subject and object. Following Gibson et al. (2013), sentence interpretation is assessed using yes‐no comprehension questions. The predictions of three constraints on the noise model are summarized in Table 2.

**Table 3:** A complete stimulus item for Experiment 1

Meaning		Intervening element	Order	Example
Plausible	Sentence	Aux	SO	Der Gärtner hat den Salat gegossen.
				“The gardener has watered the lettuce.”
			OS	Den Salat hat der Gärtner gegossen.
				“The lettuce, the gardener has watered.”
	Question	Goss der Salat jemanden/etwas?		
		“Did the lettuce water someone/something?”		
	Sentence	Main verb	SO	Der Gärtner goss den Salat.
				“The gardener watered the lettuce.”
			OS	Den Salat goss der Gärtner.
				“The lettuce, the gardener watered.”
	Question	Hat der Salat jemanden/etwas gegossen?		
		“Has the lettuce watered someone/something?”		
Implausible	Sentence	Aux	SO	Der Salat hat den Gärtner gegossen.
				“The lettuce has watered the gardener.”
			OS	Den Gärtner hat der Salat gegossen.
				“The gardener, the lettuce has watered.”
	Question	Goss der Gärtner jemanden/etwas?		
		“Did the gardener water someone/something?”		
	Sentence	Main verb	SO	Der Salat goss den Gärtner.
				“The lettuce watered the gardener.”
			OS	Den Gärtner goss der Salat.
				“The gardener, the lettuce watered.”
	Question	Hat der Gärtner jemanden/etwas gegossen?		
		“Has the gardener watered someone/something?”		

### 2.1 Method

#### 2.1.1 Participants

Experiment 1 tested 48 native speakers of German who were recruited over Prolific (http://prolific.co), with filter criterion “native language = German.” In addition, participants were asked to confirm at the beginning of the session that their native language was German. Participants were allowed to run the experiment on a desktop computer or a tablet. Participants were naive with respect to the purpose of the experiment. Their mean age was 36, ranging from 19 to 67. Participants received £4.50 or the equivalent amount in the currency of their country of residence for participation.

#### 2.1.2 Materials

Thirty‐two experimental sentences were created for Experiment 1, each appearing in eight conditions according to the factors *Order* (SO vs. OS), *Meaning* (plausible vs. implausible), and *Intervening Verb* (Auxiliary vs. Main Verb). All factors were varied within participants and within items. A complete item is shown in Table 3. The complete item list is provided at https://osf.io/9trcm/. Each sentence consisted of a subject NP, an object NP, and a main verb. The main verb was either inflected for past tense or occurred as a past participle together with a finite form of the perfect auxiliary *haben* (“have”). All subject and object NPs consisted of a definite determiner and a male singular noun. NPs of this type are unambiguously marked for case on the determiner, either *der* (“the‐nom”) or *den* (“the‐acc”). The factor Order varied whether the subject preceded the object (SO) or the object preceded the subject (OS). The factor Intervening Verb varied whether the finite main verb (past tense) or the finite auxiliary (perfect tense) followed the clause initial NP. In perfect tense sentences, the main verb was in clause final position. Sentences had a plausible meaning when the verb was combined with an animate subject and an inanimate object. Implausible sentences were derived from plausible sentences by switching subject and object. We confirmed the validity of our assumptions concerning the effect of our experimental manipulations on the prior probability by computing probabilities for all sentences in each condition using a large language model (see https://osf.io/9trcm/ for details). OS sentences were found to be less likely than SO sentences, and implausible sentences were less likely than plausible sentences, as expected.

For each sentence, a yes‐no question was created. All questions had SO order and only varied according to the factor Plausibility. For one half of the experimental items, the question had to be answered “yes,” for the other half, the answer was “no.” For the example in Table 3, the answer is “no” (see Table 5 for an example with answer “yes”). When the answer was yes, the subject of the experimental sentence was also the subject of the question. This resulted in a plausible question for plausible sentences and an implausible question for implausible sentences. When the answer was “no,” the subject of the question was the object of the experimental sentence. In this case, plausible sentences were paired with implausible questions and implausible sentences with plausible questions.

**Table 4:** Mixed‐effects model for the accuracy results of Experiment 1

Effect	Estimate	Est. Error	L‐95% CI	U‐95% CI	$p(\hat{b}>/<0)$
Intercept (SO plausible)	5.14	0.49	4.24	6.18	1
Meaning/Implausible	−1.04	0.53	−2.08	0.01	.974
**Order/OS**	**−1.06**	**0.50**	**−2.04**	**−0.07**	**.981**
Verb	0.11	0.56	−0.98	1.20	.580
**Meaning/Implausible** × **Order/OS**	**−1.10**	**0.53**	**−2.14**	**−0.06**	**.981**
Meaning/Implausible × Verb	0.11	0.60	−1.08	1.28	.580
Order/OS × Verb	−0.83	0.61	−2.04	0.37	.913
Meaning/Implausible × Order/OS × Verb	−0.50	0.66	−1.80	0.80	.771

*Note*. Effects are considered to have unequivocal evidence when 0 is not contained in the credible interval and the probability of the effect being in a particular direction is above .975. Effects for which this holds are marked in boldface. Abbreviation: *CI*, credible interval.

**Table 5:** A complete stimulus item for Experiment 2

Meaning		Order	Example
Plausible	Sentence	SO	Der Kapitän hat den Felsen übersehen.
			“The captain overlooked the rock.”
		OS	Den Felsen hat der Kapitän übersehen.
			“The rock, the captain overlooked.”
		Passive	Der Felsen wurde vom Kapitän übersehen.
			“The rock was overlooked by the captain.”
	Question	Hat der Kapitän jemanden/etwas übersehen?	
		"Did the captain overlook someone/something?"	
Implausible	Sentence	SO	Der Felsen hat den Kapitän übersehen.
			“The rock overlooked the captain.”
		OS	Den Kapitän hat der Felsen übersehen.
			“The captain, the rock overlooked.”
		Passive	Der Kapitän wurde vom Felsen übersehen.
			“The captain was by overlooked the rock.”
	Question	Hat der Felsen jemanden/etwas übersehen?	
		"Did the rock overlook someone/something?"""	

In addition to the 32 experimental sentences, 64 filler sentences were created. The filler sentences had a similar structure and a similar length as the experimental sentences but also introduced some structural variation. Fifty filler sentences were plausible and 14 were implausible. The experimental and the filler sentences were randomized and combined in such a way that at least one filler sentence intervened between two experimental sentences. Experimental sentences were presented according to a Latin square design, such that each participant saw only one version of each sentence and an equal number of sentences per condition. Thus, each participant saw a total of 96 sentences, of which 66 (16 experimental, 50 filler) were plausible (69%) and 30 (16 experimental, 14 filler) were implausible (31%).

#### 2.1.3 Procedure

The experiment was conducted online using PCIbex farm (Zehr & Schwarz, 2018). At the beginning of each session, participants read an instruction page and completed a simple form collecting demographic data including age and L1. Participants were also asked to give informed consent to participate in the study.

The procedure closely followed Gibson et al. (2013). All sentences were presented individually, along with a yes/no comprehension question designed to assess sentence interpretation. Participants were asked to answer the question by clicking on one of two radio buttons labeled “ja” (yes) and “nein” (no). The stimulus sentence, comprehension question, and answer buttons were displayed simultaneously on the same page and remained visible until the participants had answered the comprehension question and clicked on a button to move on to the next sentence. Participants were allowed as much time as they needed to complete each trial but could not return to a trial after having clicked the proceed button. Before starting the reading task, participants received three practice trials. Median session time was 14 min.

### 2.2 Results

All results presented in this paper were analyzed using the R statistics software version 4.3.2 (R Core Team, 2023). We fit a Bayesian generalized mixed model with a logit link function to the accuracy data, using the brms package (Bürkner, 2017; version 2.20.4). All experimental factors as well as their interactions were entered into the model as fixed effects. We used treatment coding for the fixed effects of Structure and Meaning with plausible SO sentences as baseline. For plausible SO sentences, a very high accuracy was expected given previous research on agent‐patient naming in German (Bader and Meng, 2018) and noisy‐channel inferences in English (Gibson et al., 2013). By taking these sentences as baseline, treatment coding provides direct evidence concerning the expected simple effects of Meaning and Structure (Brehm & Alday, 2022). The factor Verb was sum coded because there is no prior evidence that would justify taking either of the two conditions as a baseline. Participants and items were entered into the models as random effects that included the full factorial design.

We used mildly informative priors, which allow a wide range of parameter values a priori. Because the intercept corresponds to plausible SO sentences, for which yes‐no questions were expected to be answered with very high accuracy, we set the prior for the intercept to a normal distribution with a mean of 2.5 and a standard deviation of 3. This means that we could be 68% certain that the intercept would fall between −0.5 and 5.5 on the log‐odds scale, which approximates an interval ranging from 37.8 to 99.6 in terms of percentages. The priors for the effects and interactions, the standard deviations, and the correlations were set to the standard values provided in the literature (e.g., Avetisyan et al., 2020; Cutter et al., 2024; Christianson et al., 2023; Dempsey et al., 2023). Four sampling chains were run for the model, each with 12,500 iterations with one fifth of these being warmup. R Scripts and data can be found at https://osf.io/9trcm/.

**Fig. 1:**
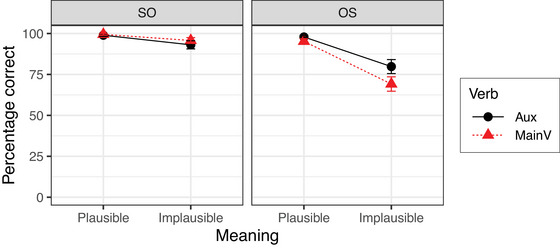
Percentages of correct answers in Experiment 1. Error bars show 95% confidence intervals.

Fig. 1 shows the mean accuracy for answering yes‐no questions in Experiment 1. The results of the Bayesian mixed‐effects model are summarized in Table 4. The model reveals a simple effect of Order OS, which reflects the finding that the percentage of correct answers was extremely high in the baseline condition of plausible SO sentences (99.2%) but somewhat lower for plausible OS sentences (95.5%). In addition, the model reveals an interaction between Order OS and Meaning plausible. This interaction reflects the finding that the accuracy for implausible OS sentences was about 26% lower than the accuracy for the baseline plausible SO sentences, which is substantially larger than the reduction in accuracy resulting from the two simple effects of Meaning and Order alone. There is no unequivocal evidence for any other effect or interaction.

### 2.3 Discussion

Experiment 1 yielded two main results. First, a substantial number of non‐literal interpretations was found when sentences were both implausible and occurred with OS order. Second, this was found regardless of whether an auxiliary or a main verb intervened between subject and object, and our results, therefore, argue against the Intervening Main Verb Constraint in (7). Since subjects and objects are not adjuncts, the Adjunct Constraint in (5) is also not compatible with the results of Experiment 1. The Function Word Constraint in (4), in contrast, is compatible with the results of Experiment 1.

The results also show that implausible SO sentences are resistant to exchange edits. This provides further evidence that comprehenders consider both plausibility and frequency of syntactic structures when estimating the prior probability $P(s_{i})$. With implausible OS sentences, exchanging the position of the determiners results in a sentence with plausible meaning and the more frequent SO order. Therefore, the gain in prior probability due to increased plausibility is even further increased for structural reasons. Applying the exchange operation to implausible SO sentences also results in a sentence with plausible meaning, but with less frequent OS order. Therefore, the gain in prior probability due to increased plausibility is lowered due to the change to OS order and comprehenders are less willing to adopt a nonliteral interpretation. This pattern is consistent with the predictions of the Function Word Constraint and supports the claim that comprehenders' nonliteral interpretations of implausible OS sentences are brought about by determiner exchanges as in (13) and not by noun exchanges as in (14).

The results also provided evidence that plausible OS sentences were answered with somewhat lower accuracy than plausible SO sentences. Since, as discussed above, it is highly unlikely that participants turn plausible into implausible sentences, this effect cannot be due to participants interpreting plausible OS sentences in a nonliteral way. We assume instead that the small disadvantage for OS sentences reflects the markedness of OS sentences when they are presented out of context. Effects of similar size have been found before in experiments in which accuracy is at an overall high level (e.g., Bader and Meng, 2018).

## 3 Experiment 2

Experiment 1 found that German OS sentences with low prior probability $P(s_{i})$ cause participants to give answers indicating that the sentences were interpreted in a nonliteral way. According to the Noisy Channel Model, this happened because the increase in $P(s_{i})$ due to interpreting sentences nonliterally was larger than the decrease of $P(s_{i}\to s_{p})$ caused by applying edit operations in order to arrive at a nonliteral interpretation. The finding that implausible OS sentences elicited a substantial number of nonliteral interpretations, whereas implausible SO sentences hardly did so, follows if comprehenders hypothesize that the perceived implausible sentences resulted from a determiner exchange applied to intended plausible sentences. With noun exchanges instead of determiner exchanges, implausible SO and OS sentences should have caused the same rates of nonliteral interpretations. The results of the first experiment, therefore, support the Function Word Constraint.

In order to corroborate this conclusion, Experiment 2 investigates passive sentences in addition to active SO and active OS sentences. The passive variants of the plausible and implausible SO/OS sentences discussed above are given in (22).


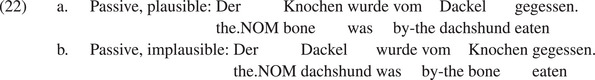


Like active SO‐ and OS‐sentences, implausible passive sentences can result when the speaker intended a plausible sentence but exchanged either nouns or function words. The exchange of nouns is shown in (23).


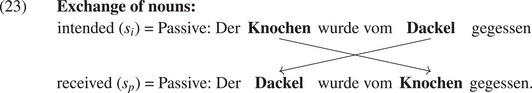


(24) shows how an implausible passive sentence results from exchanging function words.


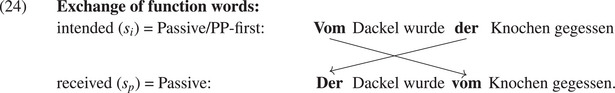


With respect to function word exchanges, there are two important differences between passive and OS sentences. First, in the case of OS sentences, the exchange involves function words of the same syntactic category, namely, two determiners (see (13)). In the case of passive sentences, on the other hand, a preposition must be exchanged with a determiner (see (24)). Word exchanges found for speech errors typically involve words of the same syntactic category (e.g., Garrett, 1975), so exchanging a preposition with a determiner is an unlikely source of corruption.

Second, as illustrated in (13), the exchange of determiners in an OS sentence results in an SO sentence. Since SO sentences are much more frequent than OS sentences, the prior probability $P(s_{i})$ of the edited SO sentence is substantially higher than the prior probability of the perceived OS sentence. For passive sentences, the reverse holds. As shown in Table 1, canonical passive sentences with the subject in initial position have a much higher prior probability than passive sentences with an initial by‐PP. Thus, applying edit operations that turn a subject‐initial passive sentence into a PP‐initial passive sentence leads to a strong decline in prior probability.

In sum, although passive sentences and OS sentences are both non‐canonical sentences, the Function Word Constraint makes different predictions for them. In comparison to OS sentences, repairing implausible passive sentences by function word exchanges costs more and gains less. It is more costly because it involves an unlikely edit operation, namely, the exchange of words of unlike syntactic category. It gains less because it decreases the prior probability of the syntactic structure, thereby reducing the gain in prior probability brought about by switching from an implausible to a plausible sentence. Thus, if the noise model used by speakers of German is subject to the Function Word Constraint, as suggested by Experiment 1, OS sentences and passive sentences should differ: in contrast to what has been found for OS sentences in Experiment 1, nonliteral interpretations should be rare or even absent for German passives, similar to what has been found for English passives. On the other hand, if German speakers allow noun exchanges, a substantial number of nonliteral interpretations should be observed for passives.

### 3.1 Method

#### 3.1.1 Preregistration

Experiment 2 was preregistered at OSF before starting data collection (https://doi.org/10.17605/OSF.IO/FQ8MD).

#### 3.1.2 Participants

Experiment 2 tested 74 participants recruited via Prolific using the same filter criteria as in Experiment 1. In addition, members of the Prolific pool who had participated in Experiment 1 were excluded. Mean participant age was 35, ranging from 20 to 73. Participants received £3.00 or the equivalent amount in the currency of their country of residence for participation.

#### 3.1.3 Materials

Thirty sentences from Experiment 1 were refactored for Experiment 2 such that every sentence appeared in six versions according to a 2 × 3 factorial design with factors *Meaning* (plausible vs. implausible) and *Structure* (active SO vs. active OS vs. passive). A complete item with all six versions is shown in Table 5. Active SO and active OS sentences were identical to the corresponding sentences in the condition “intervening auxiliary” of Experiment 1. In these sentences, the perfect auxiliary *hat* occurred between subject and object and a past participle occurred in clause final position. Passive sentences were formed by replacing the perfect auxiliary with the passive auxiliary *wurde* and by embedding the agent NP into a *von*‐PP, which corresponds to an English *by*‐PP. All passive sentences started with the subject NP (the patient argument) and the *von*‐PP (the agent argument) followed the finite auxiliary. Questions all had SO order and varied only according to the factor plausibility. Half of the experimental items had a question requiring “yes” as answer, for the other half, the answer was “no.” In the example in Table 5, the answer is “yes.” We again checked the prior probabilities of the sentence materials using a large language model. SO sentences were found to be more probable than passive sentences and passive sentences in turn to be more probable than OS sentences. In addition, plausible sentences were more probable than implausible sentences.

The 30 experimental items were distributed over six lists according to a Latin square design, as described for Experiment 1. Each list was combined with the same set of 64 filler sentences already used in the preceding experiment. Participants, therefore, saw 29 implausible sentences (15 experimental, 14 fillers) and 65 plausible sentences (15 experimental, 50 fillers), which gives a rate of 31% implausible sentences in the total sentence list.

#### 3.1.4 Procedure

The experiment was run online on PCIbex farm using the same procedure as Experiment 1. Median session  time was 14 min.

### 3.2 Results

**Fig. 2:**
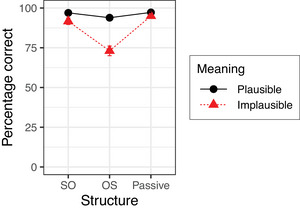
Percentages of correct answers in Experiment 2. Error bars show 95% confidence intervals.

Fig. 2 shows the mean percentages of correct answers given in Experiment 2. The results of the corresponding Bayesian mixed‐effects model are summarized in Table 6. The model shows neither a simple effect of Meaning nor any simple effect of Structure. Of the two interactions, the model provides evidence for an interaction between Meaning Implausible and Structure OS, which reflects the finding that implausible OS sentences received substantially fewer correct answers than expected given the simple effects of Meaning and Structure. The model provides no evidence for an interaction between Meaning Implausible and Structure Passive, in accordance with the finding that there was almost no difference in accuracy between plausible and implausible passive sentences.

**Table 6:** Mixed‐effects model for the accuracy results of Experiment 2

Effect	Estimate	Est. Error	L‐95% CI	U‐95% CI	$p(\hat{b}>/<0)$
Intercept	3.84	0.35	3.21	4.57	1
Meaning/Implausible	−0.52	0.41	−1.31	0.30	.900
Structure/OS	−0.60	0.38	−1.34	0.16	.941
Structure/Passive	0.34	0.42	−0.46	1.20	.791
**Meaning/Implausible** × **Structure/OS**	**−1.26**	**0.44**	**−2.14**	**−0.40**	**.998**
Meaning/Implausible × Structure/Passive	0.64	0.52	−0.37	1.69	.893

*Note*. Effects are considered to have unequivocal evidence when 0 is not contained in the credible interval and the probability of the effect being in a particular direction is above .975. Effects for which this holds are marked in boldface. Abbreviation: CI, credible interval.

### 3.3 Discussion

The main finding of Experiment 2 is that the two types of noncanonical sentences—OS sentences and passive sentences—resulted in different rates of nonliteral interpretations. As in Experiment 1, implausible OS sentences induced a substantial number of nonliteral interpretations, resulting in a robust interaction between Meaning/Implausible and Structure/OS. Implausible passive sentences, in contrast, patterned with SO sentences and were comprehended literally most of the time. This replicates studies that also failed to find evidence for misinterpretation of German passives (Grillo et al., 2018) and is consistent with the results on English passive sentences reported in Gibson et al. (2013).

The results of Experiment 2 provide additional support for the claim that comprehenders consider function word exchanges as a likely source of noise, but not noun exchanges. The results also reconfirm the interaction of prior probability $P(s_{i})$ and noise model $P(s_{i}\to s_{p})$. Implausible OS sentences can be repaired by assuming that the speaker intended a plausible SO sentence but exchanged determiners. This leads to a somewhat lower probability $P(s_{i}\to s_{p})$, but substantially increases the prior probability $P(s_{i})$ because the corresponding SO sentence has a plausible meaning and a more frequent syntactic structure. With passives, on the other hand, function word exchanges involve words of different categories (determiner vs. preposition), which is less likely compared to determiner exchanges. In addition, the gain in plausibility due to the plausible meaning of the corrected sentence is offset by the lower frequency of passives starting with a PP.

In sum, the results obtained in this and the preceding experiment suggest that noisy channel inferences are constrained in two ways. First, word exchanges are considered if they switch function words of the same syntactic category. This allows determiner exchanges but not noun exchanges, in accordance with the Function Word Constraint of Poppels & Levy (2016). Second, exchange edits are applied only when resulting in a more likely syntactic structure. This prevents function word exchanges from applying to SO and passive sentences.

## 4 Experiment 3

According to the Noisy Channel Model, a key factor in evaluating whether a perceived sentence is identical to the sentence that was intended is the prior probability of the intended sentence. The prior probability encodes (among other things) the comprehenders' world knowledge as well as their knowledge about the frequency of certain syntactic structures. In addition, the prior probability of a sentence depends on contextual factors, such as how likely it is that a sentence with implausible meaning is produced in a communicative situation. In a standard everyday situation, the probability that sentences with implausible meaning are produced is rather low, as we expect sentences to be informative, and producers of a sentence to be cooperative. However, in specific situations, such as when engaging in a language game, implausible sentences may occur with a different probability. As discussed in Gibson et al. (2013), situations in which implausible sentences are likely to be uttered should discourage comprehenders from adopting nonliteral interpretations. In this case, repairing an implausible sentence to a close alternative with a plausible meaning does not lead to a gain in prior probability because plausible and implausible sentences are equally likely to occur.

To test this prediction, Experiment 3 follows Gibson et al. (2013) and manipulates the prior probability $P(s_{i})$ by varying the rate of implausible sentences within the complete list of experimental and filler sentences presented to participants. In one condition (p15), there are 15% implausible sentences (15 implausible experimental sentences, 85 plausible filler sentences). In the other condition (p50), there are 50% implausible sentences (15 implausible experimental sentences, 35 implausible filler sentences, 50 plausible filler sentences). Under a Noisy Channel account of nonliteral interpretation, the rate of nonliteral interpretations should depend on the prior probability $P(s_{i})$. A higher rate of implausible sentences means that implausible sentences have a higher prior probability, which should decrease the number of nonliteral interpretations.

### 4.1 Method

#### 4.1.1 Preregistration

Experiment 3 was preregistered at OSF before starting data collection (https://doi.org/10.17605/OSF.IO/GQHWE).

#### 4.1.2 Participants

Experiment 3 tested 78 participants. Participants were recruited via Prolific using the same filter criteria as the preceding experiments. In addition, members of the Prolific pool who had participated in Experiment 1 or 2 were excluded. Mean participant age was 34, ranging from 21 to 59. Participants received £3.00 or the equivalent amount in the currency of their country of residence for participation.

#### 4.1.3 Materials

Fifteen experimental sentences from Experiment 2 were selected for Experiment 3. The former factor Meaning was dropped by retaining only the implausible version of each sentence. The former factor Structure (SO vs. OS vs. passive) was kept and crossed with a new factor Prior. Structure was varied within participants and items, Prior was varied between participants and within items. The 15 implausible experimental sentences were distributed across three lists according to a Latin square design. Each list was always combined with 85 filler sentences, but the number of implausible sentences within the filler set varied. In condition p15, all filler sentences were plausible, so that the total set of 100 sentences contained 15 implausible sentences. In condition p50, 35 of the filler sentences were implausible, so that the complete stimulus list contained 50 plausible and 50 implausible sentences. Each participant was randomly assigned to one of the two conditions.

#### 4.1.4 Procedure

The experiment was run online on PCIbex farm using the same procedure as in Experiments 1 and 2. Median session time was 13 min.

### 4.2 Results

**Fig. 3:**
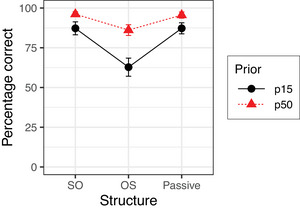
Percentages of correct answers in Experiment 3. Error bars show 95% confidence intervals.

Fig. 3 shows the mean percentages of correct answers given in Experiment 3. Table 7 summarizes the results of the corresponding Bayesian mixed‐effects model. The factor Structure was treatment‐coded as before and the factor Prior was sum‐coded. The model reveals a simple effect of Structure OS such that OS sentences resulted in lower accuracy than SO sentences, but no simple effect of Structure Passive. Furthermore, the model reveals a main effect of Prior, with higher accuracy in the condition P50 than the condition p15. This main effect was not qualified by any interaction.

**Table 7:** Mixed‐effects model for the accuracy results of Experiment 3

Effect	Estimate	Est. Error	L‐95% CI	U‐95% CI	$p(\hat{b}>/<0)$
Intercept	3.69	0.45	2.85	4.62	1
**Prior**	**1.06**	**0.53**	**0.02**	**2.11**	**.977**
**Structure/OS**	**−1.62**	**0.41**	**−2.41**	**−0.81**	**1**
Structure/Passive	0.20	0.43	−0.59	1.09	.677
Prior × Structure/OS	0.62	0.55	−0.46	1.71	.871
Prior × Structure/Passive	0.04	0.59	−1.12	1.19	.526

*Note*. Effects are considered to have unequivocal evidence when 0 is not contained in the credible interval and the probability of the effect being in a particular direction is above .975. Effects for which this holds are marked in boldface. Abbreviation: CI, credible interval.

### 4.3 Discussion

Experiment 3 found that with a low base rate of implausible sentences in the total sentence set, more errors are made when answering yes‐no questions compared to a high base rate of implausible sentences. This replicates previous findings of Gibson et al. (2013) for English and indicates that comprehenders are more inclined to derive a nonliteral interpretation for implausible sentences if implausible sentences are rare in the input, compared to a situation in which implausible sentences occur frequently.

Experiment 3 found more nonliteral interpretations not only for active OS sentences but also for active SO and passive sentences. This indicates that the lack of nonliteral interpretations in the preceding experiments was not due to inviolable constraints on edit operations. In contrast to Experiment 3, Gibson et al. (2013) did not find the semantic base rate effect for active SO and passive sentences but for other structures, including the double object construction discussed in the Introduction. Because base rate was a between‐participant factor in our and in Gibson et al.'s study, individual differences may be responsible for this difference between Experiment 3 and Gibson et al.'s study. Alternatively, this difference could be the result of cross‐linguistic differences. We must leave this issue as a topic for future research.

## 5 Experiment 4

A central finding observed in Experiments 1–3 was that comprehenders sometimes assign nonliteral interpretations to implausible active OS sentences, but make only few such errors with implausible SO and passive sentences. The overall conclusion drawn so far was that the data provide support for the claim that comprehenders consider word exchanges a possible source of noise, but that word exchanges are applied to function words of the same syntactic category only, thus preventing the exchange of *vom* and *der* that would be required in case of passives.

Two anonymous reviewers suggested an alternative account which assumes a different source of noise. According to this account, the effects observed for implausible OS sentences could also be explained if comprehenders assume deletion and insertion processes at the level of individual letters instead of whole‐word exchanges. Consider the implausible OS sentence *Den Dackel hat der Knochen gegessen* “The dachshund, the bone has eaten” from which comprehenders often erroneously recover the meaning of the plausible SO sentence *Der Dackel hat den Knochen gegessen* “The dachshund has eaten the bone.” According to the account argued for here, these errors arise because comprehenders consider it likely that the intended sentence was corrupted by a function word exchange affecting the determiners *der* and *den* (see (13)). However, it is possible that noisy channel inference in this case does not affect the word level but the letter level. Instead of hypothesizing an exchange of function words, comprehenders may arrive at the same nonliteral interpretation by hypothesizing letter edits. Hence, in the determiner *den* in *den Knochen*, the letter “n” is replaced with “r.” In the same vein, the letter “r” in the determiner of *der Dackel* is replaced with the letter “n.” According to the letter edit account, comprehenders often infer a plausible meaning from an implausible OS sentence containing definite NPs because the distance at the letter level between *den* and *der* is small, requiring the edit of a single letter only.

In case of implausible passive sentences, the letter edit distance is larger and such edits are accordingly less probable, leading to fewer errors, as found in Experiment 2. To recover a plausible meaning for the implausible passive sentence *Der Dackel wurde vom Knochen gegessen*, comprehenders would have to assume that in the received utterance *der* erroneously replaced *vom* and *vom* erroneously replaced *der*, starting from the intended sentence *Vom Dackel wurde der Knochen gegessen*. Since the distance between *der* and *vom* is three letters, such edits are predicted to be less probable, leading to fewer noisy channel inferences.

The aim of Experiment 4 is to contrast the account in terms of function word exchanges with the alternative account suggested by the reviewers in terms of letter edits and the letter edit distance. Experiment 4 is based on a proposal that one of the reviewers suggested to differentiate between the account in terms of function word exchanges versus an account in terms of letter edits. The core idea of this proposal is to use function words of the same category but with a larger letter edit distance compared to *der* versus *den*. This can be achieved, for example, if the definite article on the subject NP is replaced with an indefinite article, as shown in the examples in (25) in which the subject is combined with the definite article *der* or the indefinite article *ein*.


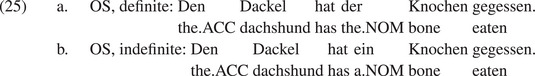


The function word account predicts that the two conditions should elicit similar rates of nonliteral interpretations. If comprehenders form nonliteral interpretations by inferring function word exchanges, it should not matter whether the subject NP contains definite *der* or indefinite *ein* since both are function words of the same category. The sentence which comprehenders infer as intended sentence is shown in (26)


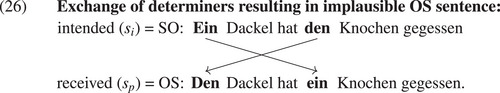


The letter edit account, on the other hand, predicts a difference. Sentences with a definite subject NP should elicit more nonliteral interpretations compared to sentences with an indefinite subject NP. According to the letter edit hypothesis, the intended sentences should look as shown in (27)


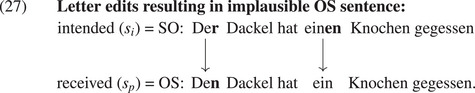


The important difference between (27) and sentences with two definite NPs as examined in the preceding experiments (and used here as controls) is that whereas *der* and *den* in the definite article condition require editing one letter only, *ein* and *einen* have a larger letter edit distance, which should lower the probability that comprehenders infer letter edits.

### 5.1 Method

#### 5.1.1 Preregistration

Experiment 4 was preregistered at OSF before starting data collection (https://doi.org/10.17605/OSF.IO/7P695).

#### 5.1.2 Participants

Experiment 4 tested 48 participants. Participants were recruited via Prolific, with filter criterion “native language = German.” In addition, participants were asked to confirm at the beginning of the session that their native language was German. Mean participant age was 37, ranging from 23 to 73. Participants received £3.50 or the equivalent amount in the currency of their country of residence for participation.

#### 5.1.3 Materials

Experiment 4 tests German active sentences with implausible meaning that varied along two dimensions: whether the subject was a definite or indefinite NP, and whether sentences showed SO or OS order. This resulted in a 2 × 2 factorial design with factors *Determiner* (same vs. different) and *Structure* (SO vs. OS). Both factors are varied within participants and items.

Twenty experimental sentences from Experiment 2 were selected for Experiment 4. The former factor Meaning was dropped and only the implausible version of each sentence was retained. The former factor Structure was reduced to the two levels SO and OS. A new factor Determiner was created with the two conditions “same” and “different.” The condition “same” of the factor Determiner had a definite determiner for both subject and object, as in the preceding experiments. In the condition “different,” an indefinite determiner was used for the subject and a definite determiner for the object. The 20 implausible experimental sentences were distributed across four lists according to a Latin square design. Each list was combined with 80 filler sentences, which were all plausible. The percentage of implausible sentences was thus 20%. Each participant was randomly assigned to one of the four stimulus lists.

Questions all had SO order. Half of the experimental items had a question that must be answered “yes,” for the other half, the answer was “no.”

#### 5.1.4 Procedure

The experiment was run online on PCIbex farm using the same procedure as in Experiments 1 through 3. Median session time was 15 min.

### 5.2 Results

**Fig. 4:**
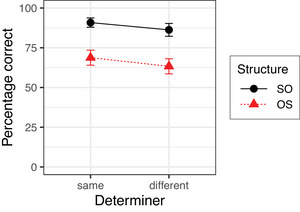
Percentages of correct answers in Experiment 4. Error bars show 95% confidence intervals.

Fig. 4 shows the mean percentages of correct answers given in Experiment 4. Table 8 summarizes the results of the corresponding Bayesian mixed‐effects model. The factor Structure was treatment‐coded as before and the factor Determiner was sum‐coded. The model reveals a simple effect of Structure OS such that OS sentences resulted in lower accuracy than SO sentences, but neither a main effect of Determiner nor an interaction between Structure and Determiner.

**Table 8:** Mixed‐effects model for the accuracy results of Experiment 4

Effect	Estimate	Est. Error	L‐95% CI	U‐95% CI	$p(\hat{b}>/<0)$
Intercept	3.61	0.42	2.83	4.48	1
Determiner	0.46	0.39	−0.31	1.22	.833
**Structure OS**	**−2.29**	**0.32**	**−2.93**	**−1.65**	1
Determiner × Structure OS	−0.13	0.45	−1.01	0.76	.612

*Note*. Effects are considered to have unequivocal evidence when 0 is not contained in the credible interval and the probability of the effect being in a particular direction is above .975. Effects for which this holds are marked in boldface. Abbreviation: CI, credible interval.

### 5.3 Discussion

Experiment 4 contrasted the function word account of noisy channel inferences for noncanonical sentences with an account in terms of letter edits. Both accounts ascribe errors with implausible OS sentences to noisy channel inference but differ regarding the source of noise they assume to guide the inference process. According to the function word account, comprehenders assume function word exchanges, but only if the function words are of the same category, which explains why comprehenders interpret implausible passive sentences in a literal way most of the time. According to the account in terms of letter edits, comprehenders assume letter replacements, which they consider probable if only one letter needs to be edited (as with implausible OS sentences), but less probable if more letter edits are required (as with implausible passives).

Experiment 4 showed that implausible OS sentences elicit a substantial number of nonliteral interpretations, regardless of whether subject and object were both definite NPs (same condition) or whether determiners differed because the subject NP was indefinite. The lack of an effect of Determiner is consistent with the function word account, since definite and indefinite determiner are of the same category, hence should trigger function word exchanges with similar probability. In contrast, the letter edit account predicted that implausible sentences containing an indefinite subject NP (indefinite condition) should significantly reduce the number of nonliteral interpretations. The results of Experiment 4 provide no evidence for such a reduction. Note that a letter edit account is also challenged by the finding of Poppels & Levy (2016) discussed above that implausible double PP sentences in English often elicit misinterpretations although the letter edit distance between *from* and *to* is rather large (see (6)).

## 6 Experiment 5

The preceding experiments converge on the conclusion that comprehenders consider the exchange of words to be a likely source of noise if function words of the same syntactic category are exchanged, but not if the exchange involves nouns or function words of different syntactic categories (e.g., preposition and determiner). Like most prior work on noisy channel inference, Experiments 1–4 assessed sentence comprehension by means of yes‐no questions. Using this procedure, the edit operations used when comprehending sentences of low prior probability cannot be observed directly but must be inferred from the ensuing error patterns. Following Ryskin et al. (2018), we will call corrections inferred from answers to yes‐no comprehension questions implicit corrections.

Ryskin et al. (2018) took a different approach to assess noisy channel inferences. Instead of yes‐no comprehension questions, their study used an explicit, free‐form correction task. Participants were given 95 sentences, which were claimed to be transcripts of someone's speech. Participants were told that some of the sentences might contain errors and they were asked to correct sentences that they thought were not encoding what the speaker had intended. The experimental sentences included three different types of errors—errors resulting from a deletion (*The uncle sold the truck the father*), from an insertion (*The earthquake shattered from the house*.), or from a noun exchange (*The oven cleaned the grandmother*). Note that Ryskin et al.'s noun exchange sentences are the English counterpart of our active SO sentences.

Two findings of Ryskin et al. are of particular importance in the current context. First, in almost all cases, the experimental sentences, which were all implausible, were corrected in one way or the other. That is, participants had no problems in spotting implausible sentences. Second, exchange edits were applied most often, and mostly by exchanging nouns. This holds in particular for simple implausible active sentences. Thus, a sentence like *The oven cleaned the grandmother* was corrected most of the time to *The grandmother cleaned the oven*. This is in contrast to the finding of Gibson et al. (2013) that implausible active sentences are rarely interpreted non‐literally, which suggests that noun exchanges hardly ever occur. The different patterns obtained by Ryskin et al. (2018) and Gibson et al. (2013) indicate that noisy channel inferences are strongly dependent on the experimental task, in particular whether the task elicits implicit corrections or requires explicit corrections as part of the task.

The task dependence of noisy channel inference making is an underexplored topic. In particular, it is an open question whether participants deploy the same noise model for tasks requiring explicit corrections (as triggered by the free‐form correction task) compared to tasks in which corrections are applied implicitly, as with answering yes‐no comprehension questions (Paape, 2024; Qian & Levy, 2023; Ryskin et al., 2018). So far, implicit and explicit corrections have been investigated in separate experiments. It is, therefore, possible that seeming task‐related differences are in fact due to differences between participants. To address this issue and to provide further evidence on the relationship between implicit and explicit corrections, Experiment 5 combines yes‐no comprehension questions with a correction task similar to the one used by Ryskin et al. (2018). Participants in Experiment 5 not only have to answer yes‐no questions, but are also required to edit implausible sentences to turn them into plausible sentences. Comparing the edited sentence with the original sentence shows directly what kind of edit operation participants apply to restore plausibility. Given the dual‐task design of Experiment 5, any differences between implicit corrections as revealed by answering yes‐no questions and explicit corrections as revealed by the correction task cannot be attributed to individual differences.

The setup of Experiment 5 differs from the preceding experiments as well as from the free‐form correction task used by Ryskin et al. (2018) in several ways. First, whereas the instructions did not give any information about the stimuli in prior experiments, in the current experiment, the instruction told participants that the experiment includes implausible sentences in order to explain the additional correction task. In the experiment of Ryskin et al. (2018), participants only did the correction task and they had to decide themselves which sentences were in need of an edit. In Experiment 5, in contrast, participants answer a yes‐no comprehension question for all sentences, and are explicitly prompted to provide a correction for all implausible sentences. Because participants in Ryskin et al.'s were highly successful in detecting implausible sentences, we do not think that prompting participants to correct implausible sentences has any detrimental effects on the outcome of Experiment 5.

To derive hypotheses, we follow Ryskin et al. (2018) who assume that “the same noise model is at play during implicit and explicit corrections.” Based on this premise and the results of Experiments 1–4, we predict that both the response pattern observed with yes‐no comprehension questions (involving implicit correction) and with the correction task (involving explicit correction) are consistent with the Function Word Constraint. For yes‐no questions, we expect that implausible OS sentences often lead to errors, whereas implausible SO and passive sentences are comprehended with high accuracy, replicating the pattern of Experiments 1–4. Regarding the type of edits applied when corrections are explicitly required, we expect that both implausible SO and OS sentences are predominantly corrected using function word exchanges. Passive sentences, however, are expected to elicit more noun exchanges, since an exchange of function words would involve words of different categories.

Independent of any syntactic constraints, function word exchanges are expected to be the preferred means for correcting SO and OS sentences if participants prefer edits that are most economical. In Experiment 5, the sentence to correct is displayed in an editable text field so that the participants do not have to type it in completely. To swap the articles *der* and *den*, it is, therefore, sufficient to replace the “r” with an “n” and the “n” with an “r.” Swapping the nouns, on the other hand, requires significantly more use of the keyboard and/or mouse. Thus, for someone who wants to complete the experiment with least effort and as quickly as possible, function word exchanges are the best way to proceed.

### 6.1 Method

#### 6.1.1 Preregistration

Experiment 5 was preregistered at OSF before starting data collection (https://doi.org/10.17605/OSF.IO/Q2VKB).

#### 6.1.2 Participants

Experiment 5 tested 36 participants recruited via Prolific using the same filter criteria as the preceding experiments. Members of the Prolific pool who had participated in Experiment 1, 2, or 3 were excluded. Mean participant age was 37, ranging from 20 to 68. Participants received £4.50 or the equivalent amount in the currency of their country of residence for participation.

#### 6.1.3 Materials

Experiment 5 reused the materials of condition *p15* in Experiment 3. The 15 experimental sentences, which all had an implausible meaning, varied with respect to their syntactic structure (SO, OS, passive). The 15 experimental sentences were interspersed with 85 filler sentences that all had a plausible meaning.

#### 6.1.4 Procedure

The experiment was run online on PCIbex farm. The procedure was identical to the procedure of the preceding experiments except for an additional correction task. For implausible experimental sentences, participants were asked to provide a corrected version of the sentence once they had answered the yes‐no question. When they clicked on the continue button, the sentence was displayed again in an editable box. Sentences could be modified using the mouse and/or the keyboard. Participants were instructed to apply modifications that came to their mind first. After they had corrected the sentence, participants clicked on a button to proceed to the next trial.

### 6.2 Results

#### 6.2.1 Yes‐no comprehension questions

**Fig. 5:**
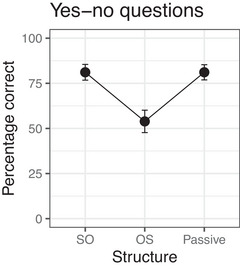
Percentages of correct answers in Experiment 5. Error bars show 95% confidence intervals.

Fig. 5 shows the mean accuracy rates for answering yes‐no questions in Experiment 5. The corresponding Bayesian mixed‐effects model is summarized in Table 9. The factor Structure was treatment coded as in the preceding experiments. The model reveals a simple effect of Structure OS but not of Structure passive. This replicates the findings of Experiments 2 and 3 that OS but not passive sentences are interpreted nonliterally more often than SO sentences.

**Table 9:** Mixed‐effects model for the accuracy results of Experiment 5

Effect	Estimate	Est. Error	L‐95% CI	U‐95% CI	$p(\hat{b}>/<0)$
Intercept	2.22	0.43	1.42	3.12	1
**Structure/OS**	**−1.74**	**0.39**	**−2.48**	**−0.94**	**1**
Structure/Passive	0.18	0.38	−0.53	0.95	.323

*Note*. Effects are considered to have unequivocal evidence when 0 is not contained in the credible interval and the probability of the effect being in a particular direction is above .975. Effects for which this holds are marked in boldface. Abbreviation: CI, credible interval.

#### 6.2.2 Corrections

The corrections that participants provided for implausible sentences were scored according to four different categories. Corrections were scored as determiner exchange if implausible sentences were turned into plausible sentences by exchanging the determiners of the two NPs. This turns SO sentences into OS sentences, and OS sentences into SO sentences. With passives, a subject‐initial passive sentence would be turned into a PP‐initial passive sentence. Corrections were scored as noun exchanges if participants switched the nouns of the two NPs to restore plausibility, thus keeping the original syntactic structure. Determiner and noun exchanges are illustrated below.


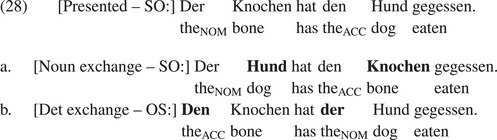


**Fig. 6:**
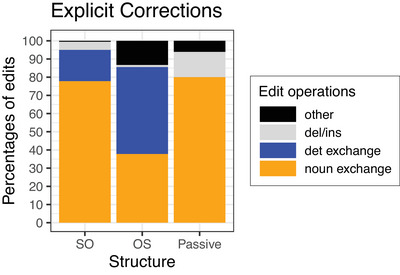
Percentages of edit operations for each syntactic structure in Experiment 5.

Corrections were scored as Insertion/Deletion if participants deleted words and replaced them with different words, for example, turning an implausible passive sentence into a plausible active sentences by deleting the passive auxiliary and the preposition. Corrections were categorized as Other if none of the three categories discussed so far applied. Fig. 6 shows how often participants used each type of edit operation for correcting each of the three syntactic structures in Experiment 5. Noun exchanges were the most frequent edit type for SO sentences (78%) and passive sentences (80%), but occurred much less frequently for OS sentences (38%). Table 10 summarizes a Bayesian mixed‐effects model with noun exchange as the dependent variable. There is no evidence for a difference between SO and passive sentences, but decisive evidence that the rate of noun exchanges is lower for OS sentences than for SO sentences. For OS sentences, determiner exchanges occurred with the highest frequency (48%). A small number of determiner exchanges were also found for SO sentences, whereas they were absent for passive sentences. The remaining edit operations occurred only rarely. An analysis of edit operations depending on whether the yes‐no question was answered correctly or not did not reveal any major differences.

**Table 10:** Mixed‐effects model for the correction task results of Experiment 5

Effect	Estimate	Est. Error	L‐95% CI	U‐95% CI	$p(\hat{b}>/<0)$
Intercept	2.34	0.60	1.20	3.55	1
**Structure/OS**	**−2.94**	**0.55**	**−3.98**	**−1.84**	**1**
Structure/Passive	0.70	0.53	−0.30	1.80	.912

*Note*. Effects are considered to have unequivocal evidence when 0 is not contained in the credible interval and the probability of the effect being in a particular direction is above .975. Effects for which this holds are marked in boldface. Abbreviation: CI, credible interval.

### 6.3 Discussion

For answering yes‐no questions, Experiment 5 replicates the results of Experiments 1–3. Questions for implausible SO and passive sentences were answered correctly most of the time, but questions for implausible OS sentences were answered correctly in only 53.9% of all cases. This high rate of nonliteral interpretations is in accordance with the low rate of 15% implausible sentences in the total sentence set. It shows that nonliteral interpretations also occur when participants are informed in advance that they will be presented with implausible sentences.

For the explicit correction task, Experiment 5 found that implausible SO and passive sentences were corrected most of the time by exchanging nouns, thereby maintaining the original syntactic structure (see (28) for SO; passive and OS work analogously). This replicates the results of Ryskin et al. (2018). Implausible OS sentences, in contrast, were predominantly corrected by exchanging determiners, which causes the edited sentence to have an SO structure. However, determiner exchanges did not predominate for OS sentences to the same degree as noun exchanges predominate for SO and passive sentences, because noun exchanges also occurred in a substantial number of cases, retaining the original OS order.

Experiment 5 is the first experiment combining a task involving implicit edits (answering yes‐no questions) with an explicit correction task. For active SO and passive sentences, which have been previously investigated in separate experiments with both tasks, Experiment 5 replicates seemingly contradictory findings in a single experiment. When answering yes‐no questions, participants eschew noun exchanges and, therefore, give few answers corresponding to nonliteral interpretations. In contrast, when corrections are explicitly required, a high proportion of noun exchanges is observed. This is all the more remarkable given that the effort involved in manually swapping nouns using a keyboard or mouse is significantly greater than swapping determiners. The choice of edit operations is, therefore, not guided by simple economic considerations.

Since the discrepancy between answering yes‐no questions and explicit corrections was observed in a single experiment, we can exclude the possibility that we are dealing with a difference between participants. The question is thus what prevents participants from applying noun exchanges to implausible SO and passive sentences when answering yes‐no questions. A further question is why OS sentences, for which no prior results are available, led to more determiner exchanges than noun exchanges when explicit corrections were required. We will consider these questions in the General Discussion.

## 7 General discussion

The experiments presented in this paper revealed a range of new findings on noisy channel inferences during language comprehension. All experiments used yes‐no questions to probe comprehension, following much prior work testing the Noisy Channel Model (Chen et al., 2023; Gibson et al., 2013; Poppels & Levy, 2016; Ryskin et al., 2025; Zhan et al., 2023; Poliak et al., 2024). For active SO and passive sentences, our experiments replicated prior findings for English. As for their English counterparts, implausible German SO and passive sentences did not result in nonliteral interpretations when the filler sentences also contained a certain number of implausible sentences. When the filler sentences did not contain any implausible sentences at all and the rate of implausible sentences was, therefore, low, a small number of nonliteral interpretations was observed even for implausible SO and passive sentences.

These findings are compatible with the conclusion that word exchanges have low probabilities in participants' noise models. For active OS sentences, however, a different conclusion emerged. For implausible active OS sentences, all experiments revealed a substantial number of nonliteral interpretations. This was true even when the rate of implausible sentences in the total sentence set was high, with a further increase in the rate of nonliteral interpretations when the rate of implausible sentences was low. As we have argued throughout this paper, the observed difference between SO and passive sentences on the one hand and OS sentences on the other hand follows if participants consider determiner exchanges but not noun exchanges as a likely source of noise.

This conclusion is further strengthened by the results of Experiment 4, which demonstrated that the rate of nonliteral interpretations is not reduced if sentences contain different determiners for subject and object. This finding is predicted by an account in terms of word exchanges, but not by a possible alternative account in terms of a simple string or letter edit process. However, the role of perceptual similarity and frequency of words affected by an exchange in noisy channel inferences should be examined more closely by future research.

Experiment 5 went beyond the first four experiments by combining yes‐no questions with an explicit correction task. For yes‐no questions, Experiment 5 replicated the results of the preceding experiments—a high rate of non‐literal interpretations for implausible OS sentences contrasts with a low rate for implausible SO and passive sentences. For the explicit correction task, Experiment 5 replicated the results of Ryskin et al. (2018): a high proportion of corrections involving noun exchanges for SO and passive sentences. However, for OS sentences, which have not been tested with an explicit correction task before, determiner exchanges were more frequent than noun exchanges, although the latter also occurred in a substantial number of cases. The prevalence of corrections involving noun exchanges in Experiment 5 constitutes another challenge for accounts of noisy channel inference in terms of simple string or letter edit processes since the letter edit distance between the two nouns in our experimental sentences is typically greater than the letter edit distance between the (short) function words *der*, *den*, and *vom*. Our findings, therefore, indicate that with explicit corrections, participants do not simply opt for corrections that minimize effort.

Given that participants make frequent use of noun exchanges when the task explicitly asks for corrections, the obvious question is what prevents participants from exchanging nouns when required to answer yes‐no questions, as evidenced by the low rate of nonliteral interpretations for SO and passive sentences when answering yes‐no questions. In the noisy channel framework, this low rate of nonliteral interpretations indicates that the perceived sentence $s_{p}$ is more likely than any alternative intended sentence $s_{i}$. According to the Bayesian formula in (1), the likelihood of a perceived sentence is the product of its prior probability and the probability that the intended sentence got corrupted. The latter probability follows from participants' noise model, that is, participants' assumptions about what kind of corruptions are likely or not. Below, we show that the prior assigned to implausible sentences strongly depends on the task required from participants. Furthermore, we argue that the differences with regard to the prior are sufficient to explain the differences between implicit and explicit corrections. We thus follow Ryskin et al. (2018) and assume that “the same noise model is at play during implicit and explicit corrections.”

In Experiment 5, participants were told in advance that they will be presented with implausible sentences for correction. Thus, participants knew that for semantic reasons, the prior probability of the presented sentence was extremely low compared to the prior probability of alternative intended sentences with a plausible meaning. Unlike Experiment 5, Ryskin et al. (2018) did not flag implausible sentences for correction, but had participants decide whether sentences needed correction or not. Their participants had no problem detecting implausible sentences, as witnessed by the low rate of implausible sentences that were not corrected. Thus, the explicit correction task used by Ryskin et al. (2018) also caused the participants to assign very low probabilities to implausible sentences compared to plausible sentences. In sum, when corrections are explicitly required for implausible sentences, participants rate the prior probability of the perceived implausible sentences $s_{p}$ as extremely low compared to the prior probability of plausible intended sentences $s_{i}$. They will, therefore, apply edit operations (almost) always. Which edit operations are applied will solely depend on how structural frequencies modulate the prior and on the probabilities of the edit operations in the noise model.

Consider next the prior probability of perceived and intended sentences when corrections are implicit, as with yes‐no questions. In most everyday situations, implausible sentences like *The bone ate the dachshund* will have an extremely low probability, whereas their plausible counterparts have a fair chance of being produced. When encountering such an implausible sentence, the comprehender will, therefore, infer that the sentence is the result of a speech error. However, this may change when sentences are presented as part of a psycholinguistic experiment. When reading an implausible sentence in the context of an experiment, the reader may reason that the implausible sentence was presented on purpose and, therefore, assign it a prior probability that is not much lower than the prior probability of a plausible sentence. That participants in experiments indeed adjust their estimation of the prior to the experimental situation is shown by experiments manipulating the base rate of implausible sentences (Experiment 3 of Gibson et al. (2013) and Experiment 3 of the current paper).

If the difference in prior probability assigned to plausible and implausible sentences is relatively small in experimental contexts, the decision about adopting the perceived sentence or an alternative intended sentence will depend on the exact ratio between prior probability and the probabilities of different edit operations within the noise model. The results of the current experiments as well as the results of prior experiments suggest that edit operations are ranked in terms of probability as follows:


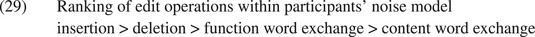


Given this ranking, explicit constraints on edit operations are not necessary. The Function Word Constraint, for which we found supporting evidence for answering yes‐no questions but not for explicit corrections, follows from function word exchanges being more likely than content word exchanges.

We first discuss experiments probing comprehension by means of yes‐no questions, that is, experiments involving implicit corrections. For English, such experiments have recurrently found that implausible sentences that can be turned into plausible sentences by a single insertion or deletion are interpreted nonliterally with some regularity (double‐object and prepositional dative sentences; see (2)), whereas implausible active and passive sentences are rarely interpreted nonliterally (see (3)). This is captured by ranking deletions/insertions higher than word exchanges, as already proposed in Gibson et al. (2013). Function word exchanges are ranked above content word exchanges to capture the finding of Poppels & Levy (2016) that double PP sentences (see (6)) result in a substantial number of nonliteral interpretations, whereas active and passive sentences do not. This ranking is also implied by the findings of the current paper, showing that implausible OS sentences are often interpreted nonliterally, whereas SO and passive sentences are much less susceptible to nonliteral interpretations.

We consider next experiments using explicit correction tasks. Noun exchanges predominate for both English and German implausible active SO sentences. For English, only noun exchanges can be used to correct SO sentences because the same determiner is used for subjects and objects, and determiner exchanges, therefore, do not have any effect. For German, in contrast, both determiner and noun exchanges can turn implausible SO sentences into plausible sentences. The strong predominance of noun exchanges can be attributed to the structural prior. While a noun exchange leaves the syntactic structure untouched, a determiner exchange causes a change from the frequent SO to the infrequent OS order (see (16) and (17)).

For passive and active OS sentences, the only data from an explicit correction task are from Experiment 5. For passive sentences, noun exchanges predominate to the same extent as for SO sentences. As for SO sentences, noun exchanges have no effect on the syntactic structure of passive sentences, whereas function word exchanges result in rather infrequent passive sentences with a sentence‐initial by‐PP (see (23) and (24)). The preference for noun exchanges with both SO and passive sentences can thus be explained in identical ways by recourse to the structural prior. In contrast to SO and passive sentences, OS sentences exhibited a slight preference for determiner exchanges over noun exchanges. In this case, a determiner exchange leads to the more frequent SO structure, whereas a noun exchange again does not change the syntactic structure. Determiner exchanges are, therefore, favored by the structural prior and, under the hypothesized ranking in (29), by the noise model. This raises the obvious question of why determiner exchanges predominate only weakly for OS sentences and why noun exchanges are also observed in a substantial number of cases for them. We hypothesize that the relatively high rate of noun exchanges for OS sentences is the result of a general bias to make as few changes as possible. Whereas the meaning and the sequence of words must necessarily be changed in order to turn an implausible into a plausible sentence, changing the syntactic structure is optional and depends on the particular edit operation. For all three structures investigated in Experiment 5, noun exchanges leave the syntactic structure unchanged and are, therefore, favored by this bias. For SO and passive sentences, a function word exchange changes the syntactic structure to a less frequent structure, leading to a strong dominance of noun exchanges. For OS sentences, in contrast, function word exchanges are favored by leading to a more frequent syntactic structure, which seems to be even somewhat more advantageous than leaving the syntactic structure untouched, resulting in only a slight predominance of determiner exchanges over noun exchanges.

Additional evidence for a bias toward retaining the original syntactic structure comes from the explicit correction experiment of Ryskin et al. (2018). Prepositional dative sentences like *The actor handed the director to the script* were most of the time explicitly corrected by a noun exchange, although a correction by deletion would also have been possible, and correction by deletion is, in fact, what is assumed by Gibson et al. (2013) for sentences of this kind. That noun exchanges nevertheless predominate is in accordance with the hypothesized bias toward leaving the syntactic structure unchanged. That this is a bias and not a hard constraint is underscored by the finding that double‐object dative sentences like *The actor handed the script the director* were corrected by an insertion in Ryskin et al.'s experiment. In this case, the cost of an insertion edit together with a changed syntactic structure is less than the cost of a noun exchange without any syntactic change.

Our study set out to test specific predictions derived from the Noisy Channel Model developed in Gibson et al. (2013). While our findings provide evidence that rational reinterpretations of the input play a role in the processing of noncanonical sentences, they do in no way exclude that other processing mechanisms contribute to misinterpretations as well, such as mechanisms designed to provide a fast route to “good enough” interpretations. As argued by Paape (2024), it is possible that the language comprehension system employs both types of mechanisms and that they affect the comprehension process to a different extent depending on syntactic construction. A task for future research is to clarify how we can determine whether a particular nonliteral interpretation is due to rational inference or to parsing errors resulting from the use of fast but fallible heuristics (see Paape, 2024 for proposals in the context of linguistic illusions).

A related question that future research needs to address concerns the role of post‐interpretive and memory‐related processes, and in particular the task dependence of noisy channel inferences. For example, while passive sentences—even with plausible meaning—gave rise to robust misinterpretation effects in studies using an agent/patient naming task (Christianson et al., 2010; Ferreira, 2003), no such effects for plausible passives, and only weak effects for implausible passives were reported in studies using yes‐no comprehension questions to assess comprehension (Gibson et al. (2013); Poppels & Levy (2016)). This suggests that passives are not affected by noisy channel inferences, but that misinterpretation effects can still be introduced by tasks that require specific memory retrievals, as argued for in Bader and Meng (2018) and Meng & Bader (2025).

## 8 Conclusion

This paper has presented five experiments that provide further evidence that comprehenders consider word exchanges as a potential source of noise in the input when processing implausible sentences, biasing them toward accepting competing alternatives with a more likely meaning. Our results suggest that exchange edits are not simple string edits but subject to structure‐sensitive constraints: exchanges are considered if they involve function words of matching category and if they result in a more frequent syntactic structure. Our results also demonstrate that constraints on exchange edits depend on the experimental task, suggesting that different tasks lead comprehenders to assign different prior probabilities to implausible sentences.

## Funding information

There was no funding for the research reported in the paper.

## Conflict of interests

The authors declare no potential conflict of interests.

## Data Availability

Data, analysis scripts, and materials of all experiments are available for downloading from the Open Science Framework: https://osf.io/9trcm/.

## Note

1
$P(s_{i}\to s_{p})$ is a notational variant of the conditional probability $P(s_{p}|s_{i})$. It is used to express in a more perspicuous way that the intended sentence $s_{i}$ is transmitted as the perceived sentence $s_{p}$.
